# Human papillomavirus type 18 infection in a female renal allograft recipient: a case report

**DOI:** 10.1186/s13256-016-1090-5

**Published:** 2016-11-09

**Authors:** Maksims Cistjakovs, Alina Sultanova, Olga Jermakova, Svetlana Chapenko, Baiba Lesina-Korne, Rafail Rozental, Dace Razeberga, Modra Murovska, Ieva Ziedina

**Affiliations:** 1 August Kirchenstein Institute of Microbiology and Virology, Riga Stradins University, Riga, Latvia; 2 Transplant Research Laboratory, Riga Stradins University, Riga, Latvia; 3 Gynecology and Maternity Unit, Pauls Stradins Clinical University Hospital, Riga, Latvia

**Keywords:** Case report, HPV-18, Renal allograft transplantation, Immunosuppressive therapy

## Abstract

**Background:**

Human papillomavirus type 18 is the second most common cause of cervical cancer and is found in 7 to 20 % of cases of cervical cancer. The oncogenic potential of high-risk human papillomavirus is associated with expression of early proteins E6 and E7. Due to long-term immunosuppressive therapy, renal transplant recipients have a higher risk of developing persistent human papillomavirus infection.

**Case presentation:**

A 29-year-old white woman from Latvia with chronic focal segmental glomerulosclerosis received renal allograft transplantation and was prescribed immunosuppressive therapy with cyclosporine, prednisolone, and mycophenolate mofetil. Two weeks after renal transplantation, her cervical swab was positive for human papillomavirus consensus sequences. After 6 months, quantitative polymerase chain reaction showed a high viral load of 3,630,789 copies/10^5^ cells of high-risk human papillomavirus type 18 and expression of E6 and E7 oncogenes in her cervical swab and urine sample. One year after renal transplantation, the viral load in her cervical swab increased significantly to 7,413,102 copies/10^5^ cells. Messenger ribonucleic acid of human papillomavirus type 18 E6 and E7 oncogenes were also detected. Shortly after this, she had an unsuccessful pregnancy which resulted in a spontaneous abortion at 6/7 weeks. Two months after the abortion her viral load sharply decreased to 39 copies/10^5^ cells. Oncogenes E6 and E7 messenger ribonucleic acid expression was not observed in this period.

**Conclusions:**

This case report represents data which show that immunosuppressive therapy may increase the risk of developing persistent high-risk human papillomavirus infection with expression of E6 and E7 oncogenes in renal transplant recipients. However, even during this therapy the immune status of a recipient can improve and contribute to human papillomavirus viral load reduction. Spontaneous abortion can be considered a possible contributory factor in human papillomavirus clearance.

## Background

Cervical cancer is the second most common cancer for women in the world. Human papillomavirus type 18 (HPV-18) is the second most common cause of cervical cancer and is found in 7 to 20 % of cases of cervical cancer [1]. The oncogenic potential of high-risk (HR) human papillomavirus (HPV) is associated with expression of early proteins E6 and E7. It has been shown that the overexpression of E6 and E7 transcripts has a leading role in the progression of the disease [2].

A clearance of HPV infection within 2 years happens in 91 % of healthy immunocompetent individuals and strongly depends on adequate immune response [3]. Due to long-term immunosuppressive therapy, renal transplant recipients have faster progression from infection to lesions and a higher risk of developing cervical cancer [4, 5].

## Case presentation

The study was approved by the Ethics Committee of Riga Stradiņš University, and written consent was obtained from the patient.

During screening for HPV in Latvian renal transplant recipients, a significant viral load of HR HPV-18 was detected in a 29-year-old white woman’s vaginal swab sample and it was taken for more detailed examination. From cervical swabs and urine, her deoxyribonucleic acid (DNA) and ribonucleic acid (RNA) were extracted for further examination (at periods of 2 weeks, 6 months, 12 months, and 16 months after transplantation). Her DNA was tested using standard polymerase chain reaction (PCR) with consensus primers targeting L1 region conservative for the majority of HPV types [6]. The commercial HPV High Risk Screen Real-TM Quant quantitative PCR (qPCR) kit (Sacace, Italy) was used for quantitative detection of 12 types of HR HPV (16, 18, 31, 33, 35, 39, 45, 51, 52, 56, 58, and 59) in her DNA samples extracted from cervical swabs and urine. Reverse transcription PCR (RT-PCR) was used to find messenger RNA (mRNA) of main HR HPV oncogenes: E6 and E7. A peripheral blood sample was centrifuged to obtain plasma for detection of immunoglobulin G (IgG) against HR HPV major capsid’s protein L1 using ELISA method (MyBioSource, USA). We also compared absolute counts of CD3, CD4, CD8, CD16, CD19, CD38, CD25, CD95 lymphocytes, and CD4/CD8 ratio progressively (6 months, 12 months, and 16 months after transplantation).

At the age of 8 years, she developed the first symptoms of a renal disease such as arterial hypertension, edema, and proteinuria. After a renal biopsy she was diagnosed with focal segmental glomerulosclerosis. In 2011 at the age of 25, she had cesarean section childbirth. After the pregnancy her renal function worsened. In March 2013 she started hemodialysis via an arteriovenous fistula in her left forearm. When she was scheduled in a waiting list for kidney transplantation (7 January 2013) she was informed and forewarned by a nephrologist about pregnancy risks and advised to use adequate contraception during immunosuppressive therapy after transplantation.

On 28 September 2013 she was admitted to a hospital to receive a renal transplantation from a deceased donor. The donor’s human leukocyte antigen (HLA) was A3,6(28); B7,44(12); DR11(5),13(6). Our patient’s HLA was A1,–; B37,39(16); DR3,D11(5). Transplantation surgery was uncomplicated and she had primary graft function.

For induction immunosuppressive therapy she received 20 mg of basiliximab (Simulect; Novartis, USA) intravenously on the day of transplantation and on the fourth day after transplantation. She was also administered methylprednisolone (Solu-Medrol; Pfizer*,* USA) intravenously for 4 days. On the day of transplantation and on the first day after the operation, she was administered 500 mg of methylprednisolone (Solu-Medrol), on the second day she was administered 250 mg, and on the third day she was administered 125 mg of methylprednisolone (Solu-Medrol).

She received prednisolone (Prednisolon; Gedeon Richter, Hungary), mycophenolate mofetil (CellCept; F. Hoffmann-La Roche, Switzerland), and cyclosporine (Sandimmun Neoral; Novartis, USA) administered orally. Her initial dosage of prednisolone was 30 mg daily and over a period of 2 weeks the dosage was gradually reduced to 20 mg per day. Her cyclosporine dosage was increased from 100 mg twice a day to 175 mg twice a day, according to through levels of 49.4 to 133 ng/ml; mycophenolate mofetil was administered 2 g daily.

**Fig. 1:**
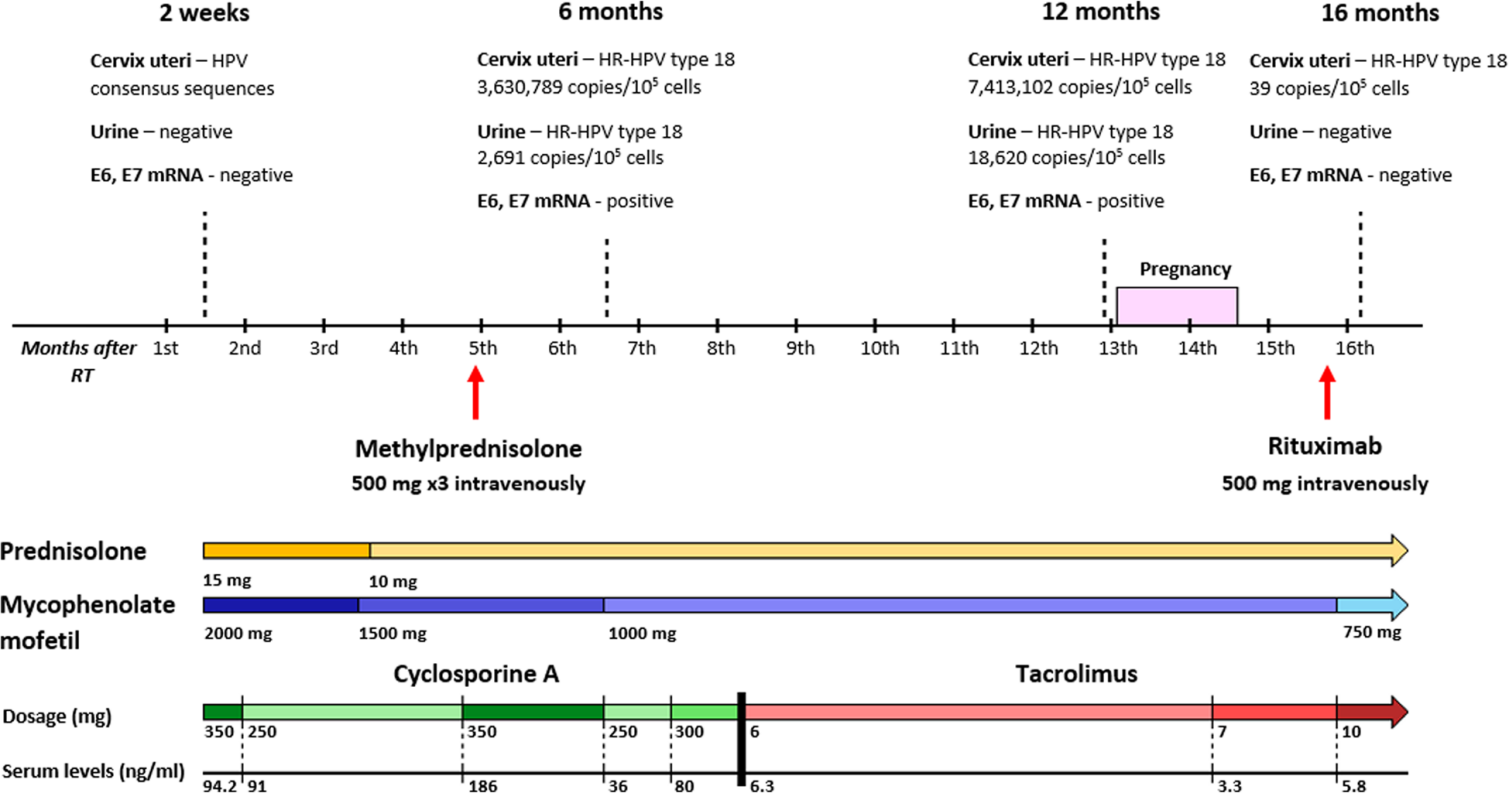
Time scale of immunosuppressive therapy and progression of human papillomavirus type 18 infection. *HPV* human papillomavirus, *HR* high-risk, *mRNA* messenger RNA

After kidney transplantation she was directed to visit a gynecologist to discuss adequate contraception during immunosuppressive therapy. She was informed once more that pregnancy is a contraindication during the first 2 years after transplantation and/or while she is receiving treatment with mycophenolate mofetil. On 14 October 2013 she was discharged from hospital with a serum creatinine level of 120 mol/l. She was prescribed the following maintenance immunosuppression therapy (Fig. 1): prednisolone, 15 mg once a day; cyclosporine, 175 mg twice a day; and mycophenolate mofetil 500 mg four times a day.

She was also prescribed 900 mg valganciclovir (Valcyte; F. Hoffmann-La Roche, Switzerland) daily for cytomegalovirus infection prophylaxis. At this point (2 weeks after surgery), a cervical swab was already positive on consensus sequences for HPV; however, there was no evidence of HR HPV infection using qPCR.

In January 2014, 3 months after her operation, she was admitted to hospital because of significant proteinuria, 1.5 g/24 hours, and increased serum creatinine level of 160 mol/l. A renal biopsy revealed T cell-mediated acute kidney rejection with borderline changes and focal segmental glomerulosclerosis of graft. She received 20 plasmapheresis sessions and 500 mg of methylprednisolone intravenously three times. Her therapy was successful and her proteinuria disappeared. Although, during this immunosuppressive therapy (6 months after operation) her qPCR results showed a significantly high load of HR HPV in a cervical swab (3,630,789 copies/10^5^ cells), but it was not significant in her urine (2691 copies/10^5^ cells). Specific typing confirmed that it was HPV-18. Expression of HPV-18 E6 and E7 oncogenes was also found in her cervical swab.

**Fig. 2:**
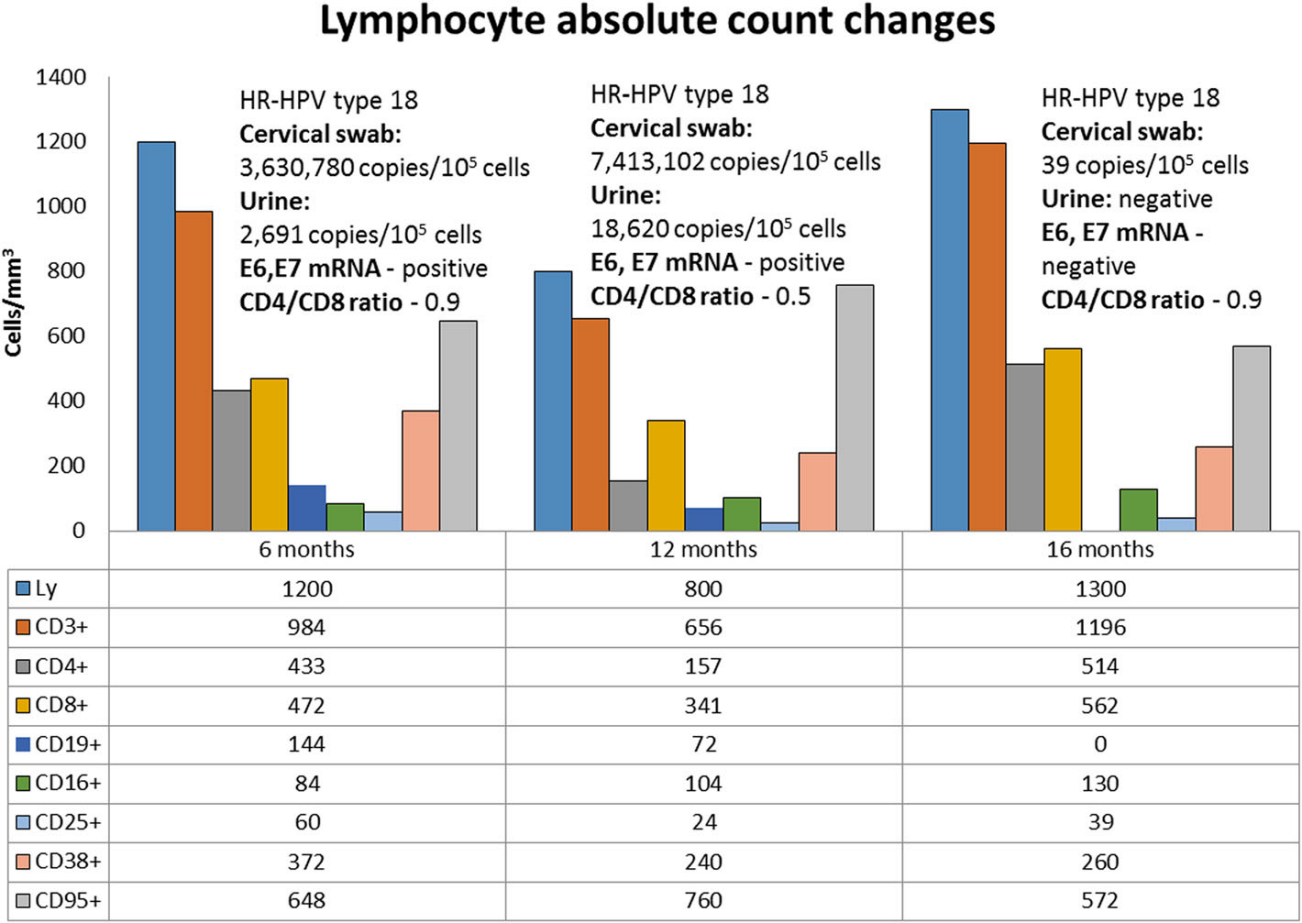
Lymphocyte absolute count changes during three periods of time (6 months, 12 months, and 16 months) depending on changes in human papillomavirus type 18 load. *HPV* human papillomavirus, *HR* high-risk, *Ly* lymphocyte, *mRNA* messenger RNA

Because of acute graft rejection, her cyclosporine was substituted with tacrolimus (Advagraf; Astellas, Japan) and her initial dose was 6 mg with serum through level of 6.3 ng/ml. During this regimen, she showed the most severe immune system suppression (Fig. 2). Exactly during this period (1 year after surgery), her HR HPV-18 viral load in cervical swab (7,413,102 copies/10^5^ cells) and urine sample (18,620 copies/10^5^ cells) doubled. Expression of HPV-18 E6 and E7 oncogenes still was detected in cervical swab.

Despite all consultations about pregnancy contraindication and all risks which were described to her by a nephrologist and a gynecologist, on 12 November 2014 she had a positive result on a human chorionic gonadotropin (hCG) hormone test for pregnancy. An ultrasound examination confirmed the presence of a gestational sac at 5 weeks of gestation. She did not tell her nephrologist in time about her pregnancy and that is why her therapy with prednisolone, tacrolimus, and mycophenolate mofetil stayed the same. At the sixth/seventh week of pregnancy she had a spontaneous abortion.

After her spontaneous abortion (December 2014) she again had a relapse of proteinuria: 1.5 g/24 hours. She received 500 mg of rituximab (MabThera; F. Hoffmann-La Roche, Switzerland) intravenously and after that she again started plasmapheresis. This time she received 15 plasmapheresis sessions. Her proteinuria decreased from 1.5 g/24 hours to 0.4 g/24 hours and she left hospital for further ambulatory observation. Two months after her spontaneous abortion, we observed a noticeable decrease in her viral load in a cervical swab (39 copies/10^5^ cells). Moreover, her urine sample did not show any viral load of HPV-18 and was negative with consensus primers for HPV sequences. Expression of E6 and E7 oncogenes was not revealed. At the same time an increase in almost all her immunocompetent cells parameters was detected (Fig. 2). During the whole study, we could not detect any IgG antibodies to HR HPV L1 major capsid’s protein in her plasma.

## Discussion

Persistence of HR HPV is strongly associated with the development of cervical cancer. In addition, several studies showed that increased viral load can also be a significant factor to predict progression to high-grade cervical lesions [7–9]. Renal allograft recipients in particular are at high risk of developing persistent infection and HPV-associated cancer [10]. Regression of HPV-associated infection requires an adequate immune response including CD4 and antigen-presenting cells (APC) infiltrate, response of T helper cells (Th1), tumor necrosis factor (TNF)-alpha production, and high CD4:CD8 ratio [11]. Immunosuppressive therapy is acting against all these factors. Prednisolone and methylprednisolone act as nonspecific inhibitors of macrophage cytokine secretion, including TNF-alpha. Cyclosporine A and tacrolimus block interleukin (IL)-2 production which is necessary for the activation and proliferation of T cells. Mycophenolate mofetil also blocks proliferation of lymphocytes by inhibiting guanine nucleotide synthesis in lymphocytes [12].

During screening for HPV in renal transplant recipients, one of the female patients stood out because she had developed a significantly elevated HR HPV load during the strengthening of immunosuppressive therapy. Initially, she was positive only on HPV consensus sequences in cervical swab and we could not find any presence of HR HPV infection neither in cervical swab nor urine sample. Six months after renal transplantation she rapidly developed HR HPV-18 infection with clinically significant viral load which we found in cervical swab (3,630,789 copies/10^5^ cells) and urine sample (2691 copies/10^5^ cells). Also, mRNA of HPV-18 E6 and E7 oncogenes were detected in the cervical swab. Such results may be explained not only by maintenance of immunosuppression therapy with prednisolone, cyclosporine A, and mycophenolate mofetil but also with recent intravenous injections of methylprednisolone, which was used to treat acute T cell rejection of the graft. After this event, she received even stronger immunosuppressive therapy (cyclosporine was substituted with tacrolimus), which was reflected by a decrease in her immunocompetent cells parameters (Fig. 2). She showed lymphopenia and her lowest counts of CD3+, CD4+, CD8+, and CD25+ lymphocyte subpopulations (Fig. 2), and CD4+/CD8+ ratio during the whole study. This could be a major factor in the doubling of her HR HPV load (7,413,102 copies/10^5^ cells) during 1 year after renal transplantation.

An impressive viral load decrease in cervical swab to 39 copies/10^5^ cells and disappearance of E6 and E7 oncogenes’ mRNA was found 1 year and 4 months after transplantation. At this time her immune status noticeably improved. Her absolute lymphocyte counts grew to normal ranges. Her count of CD3+ doubled, CD4+ tripled, CD8+ increased 1.6 times, CD25+ tripled, and CD16+ increased 1.25 times (Fig. 2). It is not fully clear what factor could influence such a sharp decrease in her HR HPV load. First, and very obvious, it could be due to her spontaneous abortion, which provoked physical damage to epithelial tissue and might have contributed to viral clearance. The literature has shown that miscarriage may be considered a significant factor that can promote clearance of HPV infection. An earlier study showed a lower prevalence of HPV DNA in females with recurrent miscarriage (26.53 %) than in females with at least one pregnancy at term (61.89 %) [13]. Also it is possible that the spontaneous abortion caused inflammation, which could explain the increase in almost all her immunocompetent cell parameters and this immune system boost could have contributed to viral clearance as well. Another factor which could be responsible for HR HPV clearance is a change in immunosuppressive therapy regimen because changes in immunosuppressive drug and dosage might improve immunocompetent cell parameters. However, the threshold for immunocompetent cell parameters to be considered to be capable to clear HPV infection is unclear.

A recent study reported that IgG antibodies to L1 capsids are essential for regression of HPV-induced cervical lesions and are associated with better prognosis [14]. However, during our research we could not detect the presence of these antibodies in our patient’s plasma. Such a result may be explained by her immunocompromised status and a lack of CD19+ lymphocyte subpopulation 12 months and 16 months after renal transplantation. This was caused by the rituximab, which inhibits activity of B cells, that she received 2 weeks before.

In the case of our patient, we observed a noticeable viral load reduction from 7,413,102 copies/10^5^ cells to 39 copies/10^5^ cells 2 months after her spontaneous abortion. It could be considered that the physical impact on cervical mucosa during spontaneous abortion and related mucosal inflammation afterwards contributed to the partial expulsion of the virus from the host. However, if virus clearance associated with lack of IgG antibodies to L1 major viral capsid’s protein is possible, then it may mean that she will have another viral load increase in the future.

## Conclusions

Immunosuppressive therapy, due to its negative efficacy on immune cell proliferation and immune activity, increases renal transplant recipients’ risk of developing persistent HR HPV infection with expression of E6 and E7 oncogenes. However, even during this therapy the immune status of a recipient can improve and contribute to a decrease in HPV viral load. Spontaneous abortion can also be considered a possible contributory factor in HPV clearance.

## Abbreviations

APC: Antigen-presenting cells
ELISA: Enzyme-linked immunosorbent assay
hCG: Human chorionic gonadotropin
HLA: Human leukocyte antigen
HPV: Human papillomavirus
HPV-18: Human papillomavirus type 18
HR: High-risk
IL: Interleukin
mRNA: Messenger RNA
PCR: Polymerase chain reaction
qPCR: Quantitative PCR
RT-PCR: reverse transcription PCR
Th1: T helper cells
TNF: Tumor necrosis factor
